# Impairment of CD4+ T and Memory B Cell Responses but Normal Memory CD8+T-Cell Activation on Crohn’s Disease after COVID-19 Vaccination: A Twin Case

**DOI:** 10.3390/v13112143

**Published:** 2021-10-24

**Authors:** Fabiana Gil Melgaço, Tamiris Azamor, Livia Melo Villar, Ana Paula Dinis Ano Bom, Juliana Gil Melgaço

**Affiliations:** 1 Laboratório de Cultu and s Biológicas, Instituto Federal de Educação, Ciência e Tecnologia do Rio de Janeiro, Duque de Caxias 25050-100, Brazil; fabiana.melgaco@ifrj.edu.br; 2 Instituto de Tecnologia em Imunobiológicos, Bio-Manguinhos, Fundação Oswaldo Cruz, FIOCRUZ, Rio de Janeiro 21040-900, Brazil; tamiris.azamor@bio.fiocruz.br (T.A.); adinis@bio.fiocruz.br (A.P.D.A.B.); 3 Laboratório de Hepatites Virais, Instituto Oswaldo Cruz, Fundação Oswaldo Cruz, FIOCRUZ, Rio de Janeiro 21040-360, Brazil; liviafiocruz@gmail.com

**Keywords:** Crohn’s disease, COVID-19, vaccine, SARS-CoV-2

## Abstract

Vaccines to prevent the impact of SARS-CoV-2 are now available, including for patients with autoimmune diseases. However, there is no information about how inflammatory bowel disease (IBD) treatment could impact the cellular and humoral immune responses. This study evaluated SARS-CoV-2-specific humoral and cellular responses after vaccination with a two-dose schedule in a Crohn’s disease patient treated with Infliximab (10 mg/kg); we included comparisons with a monozygotic twin. The results showed that the Crohn’s disease’s twin (twin 2) had no antibody detection and reduced activation of CD4+ T cell responses, unlike the twin without the autoimmune disease (twin 1). Twin 2 developed antigen-specific central memory CD8+ T-cells and IFNγ production after the second dose of COVID-19 vaccination, similar to twin 1. These findings elucidated the role of T-cell immunity after COVID-19 immunization on IBD patients despite the lack of antibody production. Finally, our observation supports the consensus recommendation for IBD patients to receive COVID-19 vaccines.

## 1. Introduction

SARS-CoV-2 has been devastating worldwide [1]. Brazil still has many cases and deaths with lower vaccination rates (11.7%) versus other countries [2]. People affected with autoimmune diseases (AID) are being urgently immunized [3], but little is known about the SARS-CoV-2 cellular immune responses on inflammatory bowel disease (IBD) patients under biological treatment [4]. A review showed that IBD patients had no worse COVID-19 symptoms versus healthy subjects [5]. Data from COVID-19-vaccinated IBD patients show controversial results along antibody detection. Some authors describe an impairment of SARS-CoV-2-IgG [6], and others reported normal detection of the specific antibodies after complete vaccination [7]. It has been known that IBD patients have an increase of pro-inflammatory cytokines, rising CD4+ helper T cells responses, mainly Th1 activation, leading to gut inflammation [8,9]. Infliximab, an anti-TNF monoclonal antibody, has been an adequate therapy for Crohn’s disease worldwide, and its effect is based on neutralizing the bioactive TNF in the intestinal mucosa, as well as it can promote apoptotic T-cell death [10]. Furthermore, CD4+ helper T cells, mainly Th2, have an important role in antibody production by B cells during viral infections or vaccination, triggering the humoral response activation and anti-inflammatory cytokines [11]. Here, we shared our findings regard to the humoral and cellular immune response in a follow-up with two monozygotic twins vaccinated for COVID-19, exploring the particularities between a healthy and an IBD twin under Infliximab therapy.

## 2. Material and Methods

### 2.1. Investigation

The twins were 37-year-old females fully vaccinated with the AstraZeneca COVID-19 vaccine. One had Crohn’s disease diagnosed in 2014 and has been on Infliximab therapy (10 mg/kg) since June 2016 administered every 8 weeks (twin 2). During the follow up, twin 2 had no signals and symptoms of gastrointestinal inflammation, and according to her latest biannual exams the Crohn’s disease was under control upon Infliximab therapy (Infliximab serum levels = 24 μg/mL; C-reactive protein = 0.28 mg/dL (normal value < 1.0 mg/dL); fecal calprotectin = 47 μg/g (normal value = 50 μg/g); serum liver enzymes: ALT = 16 U/L (normal value < 33 U/L), AST = 13 U/L (normal value < 32 U/L), ALP = 59 U/L (normal value: 35–104 U/L)).

Plasma samples were collected to assess humoral immunity by serological assays (LIAISON^®^ SARS-CoV-2 Trimerics IgG, Diasorin, Stillwater, MN, USA), and peripheral mononuclear cells (PBMC) were used for cellular immune response investigation using antigen stimulation assay by Elispot (#3420-4APT-10, Mabtech, Augustendalstorget, NS, Sweden) and assessed by flow cytometry. The subjects declared absence of COVID-19 signals and symptoms during the follow up, and reported that they had been tested by RT-PCR with nasopharyngeal swab collection (every 15 days) since March 2020 with negative results for SARS-CoV-2 detection. Twins reported no serious adverse events (Table 1).

All subjects provided their informed consent for inclusion before they participated in the study and the protocol was approved by the FIOCRUZ Ethics Committee with number: 34728920.4.0000.5262.

### 2.2. Laboratory Assays

**Figure 1:**
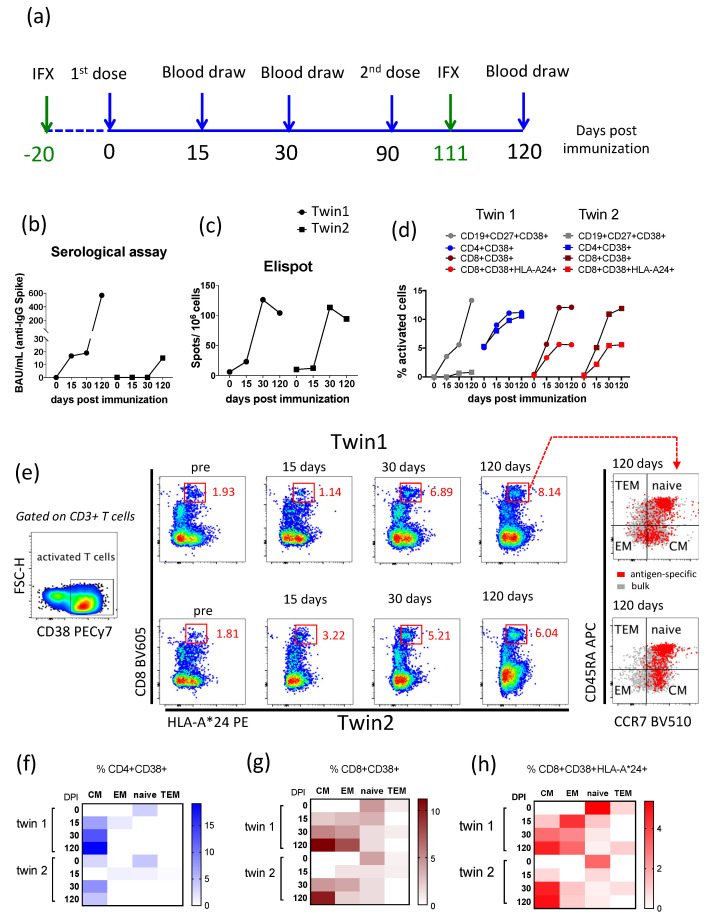
Immunological findings from the twins vaccinated against SARS-CoV-2. (**a**) The time line of the follow-up performed with monozygotic twins at days post immunization with the AstraZeneca COVID-19 vaccine, including the blood draw collection to perform immunological assays, and the days of intravenously biological treatment using infliximab (IFX) by twin 2 are highlighted in green. (**b**) Serological assay to detect specific anti-spike glycoprotein (SARS-CoV-2) antibody. (**c**) Elispot assay to detect IFNγ-secreted cells. (**d**) Percentage of polyclonal activation (CD38+) for B and T lymphocytes subsets after spike glycoprotein peptides stimulation. (**e**) Gates representing the percentage of activated CD8+ T cells expressing HLA-A*24 after antigenic stimulation over time (red chart gates), as well as those cells gated on memory subsets for both twins. Memory T cells subsets were based on CCR7 and CD45RA co-expression—TEM: terminally differentiated memory cells (CCR7-CD45RA+); CM: central memory cells (CCR7+CD45RA-); EM: effector memory cells (CCR7-CD45RA-); Naïve (CCR7+CD45RA+). (**f**) Percentage of activated CD4+ T cells after cell stimulation with spike glycoprotein. (**g**) Percentage of activated CD8+ T cells after cell stimulation with spike glycoprotein. (**h**) Percentage of activated CD8+HLA-A*24+ T cells after cell stimulation with spike glycoprotein. Baseline signals of activated cells in unstimulated controls were subtracted from SARS-CoV-2-stimulated assays to enable the visualization of SARS-CoV-2-specific B and T cells signals. Abbreviations—DPI: days post immunization. IFX: infliximab therapy.

Samples were collected at the time points described on Figure 1a and frozen at −70 °C (plasma) and in liquid nitrogen (PBMC) until the day of the laboratory assays. The polyclonal antigen-specific cellular assay was performed using defrost PBMC incubated for 48 h with supplemented RPMI (R10) containing 1 μg/mL of spike glycoprotein purchased from JPT Peptides (PepMix™ SARS-CoV-2 (Spike Glycoprotein, PM-WCPV-S, Germany) plus 20 μg/mL of spike glycoprotein [12,13].

Negative control was performed with only R10, and positive control was set up with anti-CD3 monoclonal antibody diluted 1:1000 provided by Elispot commercial kit. The surface staining to identify subpopulation of B and T cells was performed using commercial antibodies, as anti-CD3 APC-Cy7 (clone SK7), anti-CD4 Brilliant Violet 421 (clone RPA-T4), anti-CD8 Brilliant Violet 605 (clone SK1), anti-CD19 Brilliant Blue 700 (clone SJ25C1), anti-CD27 Brilliant Blue 515 (clone M-T271), anti-CD38 PECy7 (clone HIT2), anti-CD45RA APC (clone HI100), and anti-CCR7 Brilliant Violet 510 (clone 3D12), purchased from BD Biosciences. The anti-HLA-A*24 PE (clone 22E1) to identify virus-specific memory CD8+ T cell subpopulation was purchased from LSBio.

Intracellular staining (ICS) assay was also performed for cytokines detection to define subpopulations of CD4+ Th1, CD4+ Th2, and cytotoxic CD8+ T cells after 24 h with spike glycoprotein stimulation. Stimulation was performed using 10 μL/mL anti-CD28/49d (BD Biosciences, San Diego, CA, USA), and 4 μg/mL of spike glycoprotein purchased from JPT peptides, as described previously (except for the unstimulated condition) in RPMI1640 medium at a final volume of 200 μL. Furthermore, an anti-CD3 monoclonal CD3-2 was used as a positive control (Mabtech #3420-APT-2, Stockholm, Sweden). Cells were incubated for 24 h at 37 °C and 1× protein transport inhibitor (BD Biosciences, San Diego, CA, USA) was added in the last 4 h of incubation. At the end of incubation time, cells were washed and stained using a viability dye (Live/Dead Fixable Blue, #L23105, Thermo Fisher Scientific, Carlsbald, CA, USA) according to the manufacturer’s protocol. Staining was carried out using the following surface antibodies: CD3 FITC, clone: UCHT1; CD4 APC-H7, clone: RPA-T4; CD8 Brilliant Violet 605, clone: SK1; CD38 PECy7, clone: HIT2. All antibodies were purchased from BD Biosciences, San Diego, CA, USA. After surface antigen staining, cells were washed twice in FACS buffer and a fixation–permeabilization step was performed using the Cytofix & Cytoperm Plus kit ^TM^, according to the manufacturer’s protocol (BD Bioscience kit, cat#554715, San Diego, CA, USA). The following antibodies were used for intracellular staining (IFN-γ BV510, clone: B27; IL-2 Brilliant Violet 421, clone: MQ1-17H12; IL-4 Brilliant Violet 711, clone: MP4-25D2; TNF APC, clone: MAB11). All antibodies were purchased from BD Biosciences, San Diego, CA, USA. Compensation beads (UltraCompeBeads, Invitrogen^TM^, Carlsbald, CA, USA cat#01-2222-42; ArC^TM^ Armine Reactive Compensation Bead kit, Invitrogen^TM^, Carlsbald, CA, USA, cat#A10346) were used for compensation set-up.

**Figure 2:**
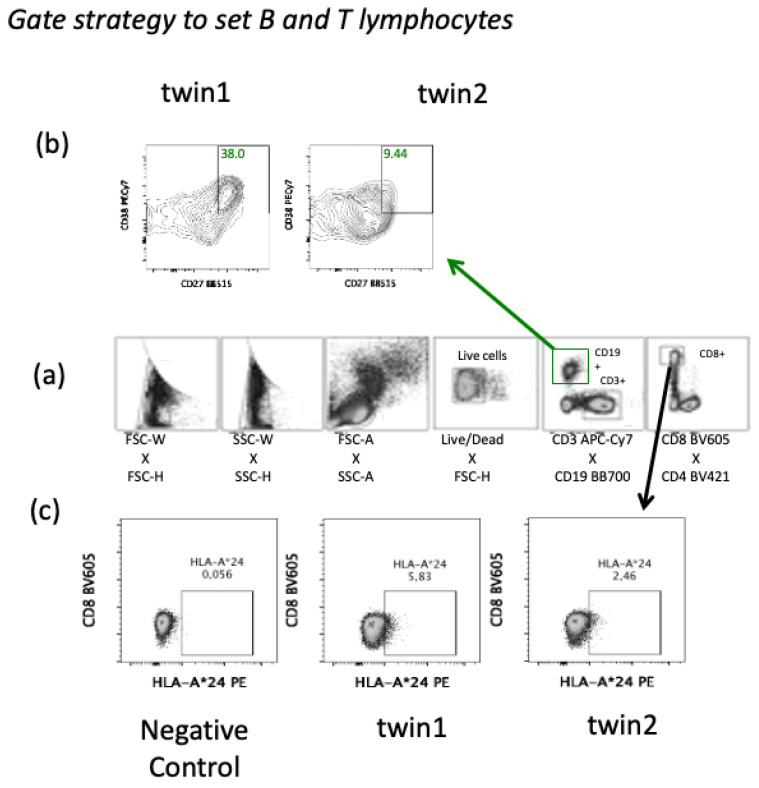
Gate strategy to set B and T lymphocytes by flow cytometry. (**a**) Gate strategy to set B cells (CD19+) (green) as well as T cells (CD3+) (black) from peripheral blood mononuclear cells. (**b**) Percentage of twins’ memory B cells subset (CD19+CD27+CD38+) activated after spike glycoprotein activation at 30 days after the second dose of COVID-19 vaccine (120 days). (**c**) Twins’ CD8+ T cells expressing the supertype HLA class I A*24 by immunophenotyping from peripheral mononuclear cells at the day 120 into the follow up (30 days after second dose of COVID-19 vaccine) and using a negative control (HLA-A*02).

The PBMC samples stained were analyzed by LSR Fortessa (BD Biosciences, San Diego, CA, USA) and FlowJo v10.7.1 (BD Biosciences, San Diego, CA, USA). Percentages of B and T cell subsets above 1% were considered for final analysis. Gate strategy to define B and T lymphocytes population is displayed in Figure 2. The unstimulated condition on ICS assay was used to subtract any background staining off the analysis. Positive ICS after spike glycoprotein stimulation was determined by detection of at least 10 SARS-CoV-2-specific T cells and a frequency of SARS-CoV-2-specific T cells of at least twice the corresponding unstimulated signal.

### 2.3. Statistical Analysis

The normality assumption of the data was initially evaluated by the Kolmogorov-Smirnov test or Shapiro-Wilk. Spearman correlation test was performed with the variables from each twin individually considering the information over time (0–120 days). The software R Studio 2021.09.0 v 4.1.1 for Macintosh was used to perform statistical analysis (https://www.r-project.org/, accessed on 11 October 2021). The significance for all statistical analyses was defined as *p* < 0.05.

## 3. Results

The absolute frequencies of B and T lymphocytes were the same between the twins except for the CD4/CD8 ratio; here, the percentage of CD19+ B cells, CD4+, and CD8+ T-cells were slightly lower in the sister with Crohn’s disease (twin 2) (Table 1). Despite the lack of great changes in the absolute frequencies on B- and T-cells, twin 2 had very low antibody detection after the completion of the COVID-19 vaccination (Figure 1b). In addition, the percentage of activated memory B cell subset (CD19+CD27+CD38+) was lower in twin 2 than twin 1 (Figure 1d and Figure 2). However, both twins had similar antigen-specific IFNγ-secreted cells detected by Elispot (Figure 1c).

The percentage of polyclonal antigen-specific activated CD4+ and CD8+ T-cells (CD38+) was similar among the twins over time even for CD8-expressing HLA-A*24 (Figure 1d). Activated memory T-cell subsets showed that central memory CD4+ was detected only at day 30 after the 1st dose for twin 2 versus twin 1, who had central memory CD4+ T-cells detectable for 15 days after the 1st dose (Figure 1f). Meanwhile, both twins had a high percentage of antigen-specific central memory CD8+ T-cells detectable after completed vaccination (Figure 1g). The specific-CD8+ T-cells expressing HLA-AI were observed in high frequency 30 days after the 1st dose. It was still high 30 days after the 2nd dose (120 days after the 1st dose) for both twins (Figure 1h). We examined memory subsets for virus-specific CD8+ T-cells expressing HLA-AI and found that effector memory CD8+ T-cells were lower, but central memory remained more frequent than other subsets of memory CD8+ T-cells in twin 2 versus twin 1 (Figure 1e).

**Figure 3:**
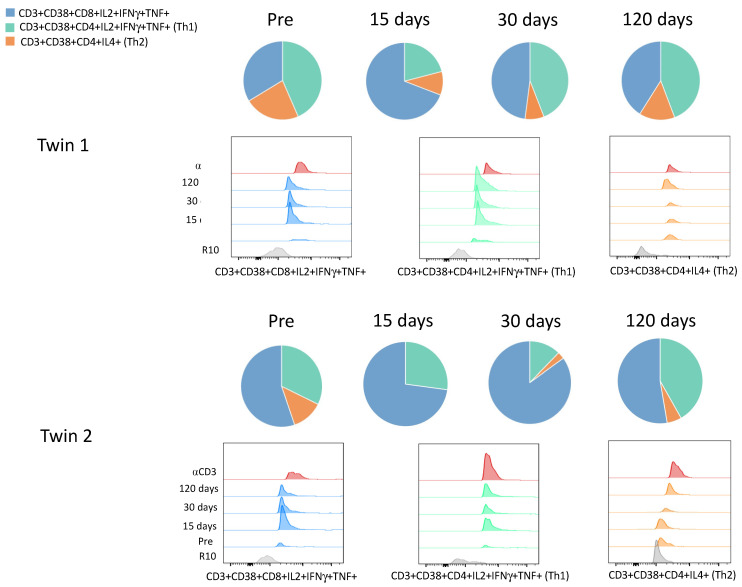
Cytokines production by CD4+ and CD8+ T lymphocytes. The pie charts show the distribution of cytotoxic CD8+ T (CD3+CD38+CD8+IL2+IFNγ+TNF+), CD4+ Th1 (CD3+CD38+CD4+IL2+IFNγ+TNF+), and CD4+ Th2 (CD3+CD38+CD4+IL4+) cells upon spike glycoprotein stimulation after vaccination for the twins over time. The histograms from FACS analysis show the expression of those cell profiles. R10: RPMI 1640 medium used as negative control for cell cultivation, αCD3: anti-CD3 monoclonal antibody used as positive control for cell cultivation. Pre: samples collected before the 1st dose of AstraZeneca COVID-19 vaccine, day 0.

When the cytokines production by T cells were evaluated, it was observed that twin 2 had similar Th1 responses (CD4+IL2+ IFNγ+TNF+) to twin 1 until 15 days but dropped after 30 days of vaccination (Table 2, Figure 3). Meanwhile, the Th2 response (CD4+IL4+) was lower in twin 2 in comparison with twin 1 (Table 2, Figure 3). Concerning TNF expression, twin 2 showed lower percentages on CD4+ and CD8+ T lymphocytes than twin 1 over time (Table 2). Moreover, the SARS-CoV-2 specific CD8+IFNγ+ was highly activated in both twins, with high activation of cytotoxic CD8+T cells (CD8+IL2+ IFNγ+TNF+) on the 15 days upon 1st dose, and it was sustained on 30 days after the 1st and 2nd doses (Table 2, Figure 3). In general, the cellular immune response kinetics were similar for CD8+ T lymphocytes on the twins, but not for CD4+ T and B-cells.

**Figure 4:**
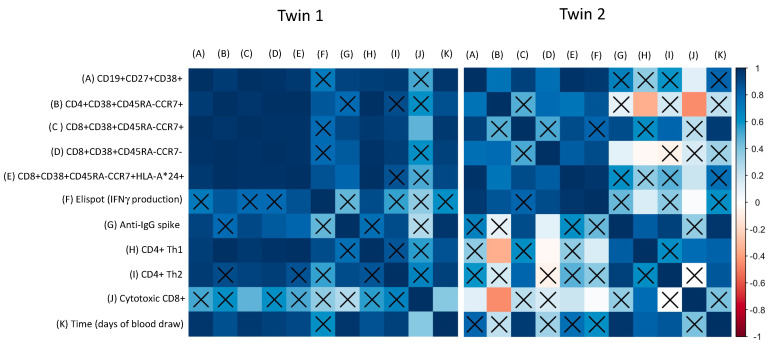
Correlation between humoral and cellular response upon COVID-19 vaccination. The correlation was calculated with individual data from the twins. (**A**) activated memory B cells, (**B**) central memory CD4+ T cells, (**C**) central memory CD8+ T cells, (**D**) effector memory CD8+ T cells, (**E**) central memory CD8+ T cells expressing HLA type, (**F**) IFNγ-secreted cells (Elispot assay), (**G**) anti-IgG spike (serological assay), (**H**) activated CD4+ Th1 cells (CD4+IL2+IFNγ+TNF+), (**I**) activated CD4+ Th2 cells (CD4+IL4+), (**J**) activated cytotoxic CD8+ T cells (CD8+IL2+ IFNγ+TNF+), (**K**) time points of blood collection. Insignificant *p* values (*p* > 0.05) are highlighted with X.

Regarding the particularities of the COVID-19 vaccine immune response observed among the twins, Figure 4 shows the correlation between the major findings detected in the study, such as the humoral response (IgG antibodies), memory B cells activation, memory T cells activation, and cytokines produced by CD8+ T and CD4+ T (Th1 and Th2) cells over time. It was noted that the healthy twin (twin 1) had positive and significant correlations between humoral and cellular responses over time, mainly for memory phenotypes for B and T lymphocytes, and effector memory CD8+ with cytotoxic CD8+ T cells (Figure 4). Therefore, it was different for the twin with IBD (twin 2), in which a negative correlation was observed between central memory CD4+ T cells and CD4+ Th1 plus cytotoxic CD8+ T cells. A positive correlation was found between IgG production and CD4+ Th2 cell response, as well as between IFNγ-secreted cells by Elispot and central memory CD8+ T cells expressing HLA plus cytotoxic CD8+ T and CD4+ Th1 cells (Figure 4).

## 4. Discussion

Vaccines to prevent the impact of SARS-CoV-2 are now available, including for patients with autoimmune diseases. However, there is limited information about how biological therapy with immunosuppressive medication will influence the immune response during COVID-19 vaccination of IBD patients [4,14]. Wellens et al. (2021) showed that humoral and cellular immune response in IBD could be impaired after some vaccines (hepatitis B, mumps, tetanus), but this was not therapy-related [15]. Wallace et al. (2014) suggest that a dysregulation of CD4+ Th1, Th2, Th17, and regulatory T cells is driven by inflammatory bowel diseases, as Crohn’s disease, which alters gut homeostasis [8]. Ricciardelli et al. (2008) showed that children with Crohn’s disease treated with Infliximab presented an expansion of mucosal regulatory T cells, which could lead to a reduction of inflammatory T cells, promoting restoration of mucosal homeostasis [16]. Noble et al. (2020) reported that IBD patients normally have an imbalance between cellular and humoral immunity in the intestinal tissue resident cells, leading to memory B-cell dysfunction [17]. Shirai et al. (2018) showed that IBD patients treated with biological therapy (e.g., Infliximab) had lower seroconversion after influenza vaccination [14]. Chebli et al. (2021) mentioned that the COVID-19 vaccine may work slightly less well in patients receiving immunosuppressors, but it is better than not having the vaccine [4]. In addition, there is no information about how IBD patients should stop their therapies to get vaccinated against SARS-CoV-2. They should avoid receiving immunosuppressive treatment on the same day of vaccination (both first and second doses) [4].

The lack of seroprotection or seroconversion in IBD patients is not directly associated with susceptibility, and the seroconversion does not translate to disease protection as in healthy subjects [15]. Unfortunately, we cannot assume that the antibody loss by the IBD twin after SARS-CoV-2 vaccination was induced by the autoimmune disease or by the biological therapy. Nevertheless, Salinas et al. (2011) suggested that anti-TNF therapy could induce an impairment of memory B cells after hepatitis B and *Streptococcus pneumoniae* vaccination through T-cell dependent humoral responses [18]. It is worthy to note that circulating CD4+ helper T cells are important to activate the follicular CD4+ helper T cells, which are crucial for induction of antibody production by B cells in the germinal center for affinity maturation and humoral memory response even after vaccination or viral infections [19,20]. Here, it was observed that Infliximab therapy regulated the Crohn’s disease, as twin 2 did not present any signal or symptoms of gut inflammation during COVID-19 vaccination, and the presence of Infliximab may have a role in the reduction/delayed of central memory CD4+ T cells, CD4+ Th1, and Th2 responses, as well as in the absence of specific IgG antibody production and memory B cell activation compared to the healthy twin (twin 1). The CD8+ cells expressing TNF were also reduced in twin 2, but the correlation between antigen-specific IFNγ-secretion by PBMC and CD8+ T cell responses suggests that memory CD8+ T cell activation was not directly affected by anti-TNF treatment. The specific IFNγ-production by CD8+ T lymphocytes seen here suggests that the AstraZeneca COVID-19 vaccine was effective in this IBD case promoting activation of the antiviral defense.

Memory CD8+ T-cells have an important role during the protective immunity after immunization. This subset with CD4+ T cell phenotypes (helper T cells and regulatory T cells) should be explored in future large studies of IBD subjects who were vaccinated. In summary, this unique case report including monozygotic twins is a good opportunity to investigate the kinetics of immune events relative to IBD. Our findings can help explain the importance of T-cell immunity relative to COVID-19 vaccination in IBD patients on biological therapy. Finally, our observation supports the consensus recommendation for IBD patients to receive COVID-19 vaccines. The finding also helps explain immunity after SARS-CoV-2 vaccination.

## Figures and Tables

**Table 1:** Absolute frequency of B and T lymphocytes and clinical information of the twins.

	Twin 1				Twin 2			
DPI	0	15	30	120	0	15	30	120
%CD19+	15.16	14.27	13.17	13.30	11.90	13.46	13.39	12.01
%CD3+	62.40	65.50	62.10	62.10	60.10	62.50	65.70	60.90
%CD4+	42.10	48.90	44.70	36.80	34.00	39.30	47.00	38.50
%CD8+	21.20	21.60	21.40	20.80	12.70	15.50	16.80	10.80
CD4/CD8	2.00	2.30	2.10	1.80	2.70	2.50	2.80	3.60
HLA-AI type	A*24:02				A*24:02			
HLA-AII type	A*68:02				A*68:02			
BMI	28.9				27.1			
AEPV								
1st dose	yes	mild fever (37.7 °C), local pain, myalgia, headache			yes	local pain, myalgia, headache		
2nd dose	no				no			

Legend: DPI: days post immunization; BMI: body mass index; AEPV: adverse events post immunization. *: it is a separator to define the international nomenclature of the HLA genotype.

**Table 2:** Percentages (%) of activated T lymphocytes subsets (CD3+CD38+) producing cytokines after stimulation with spike glycoprotein.

	Twin 1				Twin 2			
	Day 0	Day 15	Day 30	Day 120	Day 0	Day 15	Day 30	Day 120
CD4+IL2+IFNγ+TNF+ (Th1)	0.40	2.64	4.70	6.44	0.41	2.33	0.40	4.39
CD4+IL4+ (Th2)	0.21	1.10	0.85	2.14	0.23	0.01	0.09	0.58
CD4+TNF+	0.96	38.37	27.35	17.00	5.44	3.32	3.47	11.02
CD4+IL2+	0.00	0.00	13.58	15.85	0.49	3.33	0.67	1.83
CD4+IFNγ+	0.00	8.95	10.57	12.5	1.48	5.62	0.71	2.98
CD4+IFNγ+IL2+	0.00	0.00	1.82	2.83	0.00	0.22	0.01	0.04
CD4+IL2+TNF+	0.01	0.50	3.43	0.29	0.12	0.14	0.19	0.21
CD4+IFNγ+TNF+	0.00	7.56	2.30	0.48	0.40	0.61	0.00	0.83
CD8+IL2+ IFNγ+TNF+	0.51	7.70	5.11	5.98	0.83	7.09	2.77	5.53
CD8+IFNγ+IL2+	0.00	5.41	3.29	1.05	1.48	5.88	3.05	0.88
CD8+IFNγ+TNF+	0.00	5.52	4.93	5.34	0.04	1.19	0.38	0.87
CD8+IL2+TNF+	0.06	4.84	3.07	3.22	0.11	2.8	0.85	1.07
CD8+TNF+	1.20	45.46	35.97	32.9	1.12	21.38	8.30	15.21
CD8+IFNγ+	0.00	15.60	13.86	18.30	2.95	18.25	10.81	17.60
CD8+IL2+	0.12	14.33	11.00	18.29	6.39	19.21	9.73	6.68

## Data Availability

Not applicable.
